# A General Finite Beam on Tensionless Foundation Model for Rail Track Characterization and Evaluation

**DOI:** 10.3390/s26092897

**Published:** 2026-05-05

**Authors:** Hamoud H. Alshallaqi, Brett A. Story

**Affiliations:** Department of Civil & Environmental Engineering, Southern Methodist University, 3101 Dyer St Suite 105, Dallas, TX 75205, USA; halshallaqi@smu.edu

**Keywords:** tensionless Winkler foundation, finite beam, non-linear and linear stiffness behavior, varying gap, varying stiffness, iterative approach, discrete springs

## Abstract

Rail infrastructure plays an important role in freight and passenger mobility, and the assessment of rail track structure depends critically on understanding how the rail interacts with the supporting foundation. When rail support degrades (e.g., due to ballast fouling, settlement, etc.), the rail exhibits greater localized deformation that can lead to serious deleterious conditions. Track modulus represents a fundamental diagnostic measure of rail support, encompassing the vertical stiffness characteristics of the foundation and its resistance against downward rail movement. Existing track modulus characterization methodologies typically comprise deflection measurements of railway track (e.g., tie deflections) under known loads. Track modulus estimations result from analyzing deflection and load under assumptions of a traditional Winkler foundation, which can oversimplify mechanic relationships. Specifically, in the context of rail–ballast–subgrade interaction, a tensionless foundation permits gap development which can occur as track structure separates from the supporting ballast; additionally, track modulus may vary along the track length as conditions vary spatially. This paper presents a general analytical solution of ballasted track support characterization based on an iterative algorithm for the static response of a finite beam resting on a tensionless Winkler foundation. The method relates to multiple loads (e.g., concentrated axle loads and distributed self-weight), deflection along the track, and track condition through singularity functions, superposition of discrete support springs, and moment–curvature relationships. The model estimates rail deflections, lift-off points and shear and moment diagrams along the track. The technique permits: (1) validations against benchmark solutions and previously published results, (2) estimations of track modulus from known loads and measured deflections, and ultimately, (3) a framework for designing and processing sensor data streams for use in analyses and evaluations of railway track structure.

## 1. Introduction

The reliable and secure operation of railway systems promotes safe transport of both passengers and freight over approximately 140,000 miles in the United States [1]. Increased passenger and freight rail demands, heavier axle loads, and environmental factors all progressively compromise railway track integrity and can lead to the development of track-related defects, which, in turn, necessitate increased monitoring, inspection, and maintenance [2,3,4,5,6]. In the United States, derailments comprise most train accidents, and their consequences can result in significant risks, causing damage to both trains and infrastructure, service disruptions, and potentially leading to human loss and injuries or environmental harm [7]. According to the Federal Railroad Administration (FRA), track geometry defects are considered the second largest cause of derailments after broken rails [8]. Soft spots resulting in abrupt or gradual reductions in support stiffness along the rail cause the majority of derailments as a result of increased vertical deflection under load, subsequent increases in rail stresses, and increased risk of fatigue-induced fracture. This situation is exacerbated under heavy axle loads at relatively low speeds, such as typical freight operations. At higher train speeds, such as in high-speed passenger rail, abrupt stiffness variations may generate dynamic amplification of wheel–load interaction forces, which further increases loading on the track structure and accelerates deterioration [9,10,11].

Beam–foundation–load interaction models representing track support conditions provide the mechanics relationships to design, monitor and evaluate ballasted railway tracks [12,13,14,15]. Beam–foundation interaction behavior is intricate and difficult to model in full detail; therefore, engineers rely on idealized models to approximate the mechanical behavior of the beam–foundation interaction [13,16]. The Winkler model, or beam on elastic foundation (BOEF) model, assumes the railway track as an infinite beam resting on elastic supports consisting of a continuous bed of infinite independent, vertical linear elastic springs, with a spatially uniform foundation support or track modulus, *K* [13,16,17]. Despite its widespread use, the Winkler model assumes that the foundation provides identical reactions for both tension and compression, and, in many cases, this assumption does not hold (e.g., a ballasted railroad rail lifts off under certain loading conditions) [18,19]. Winkler relationships represent the prevalent mechanics relationship used in most practical assessments in both static and dynamic applications. Relying solely on the computationally efficient Winkler BOEF models can introduce errors in track modulus estimates stemming from inaccurate representation of realistic support conditions or the mismatch of dynamic vs. static or quasi-static properties. As a result, analytical predictions obtained from simplified BOEF formulations may differ from measured track responses under real operating conditions. Nevertheless, due to its conceptual simplicity and computational efficiency, the BOEF model continues to be widely adopted in railway engineering studies, including extensions that incorporate dynamic effects [20,21,22].

In light of the limitations of the Winkler model, tensionless models have been introduced, and the earliest work reported on infinite beams resting on a tensionless foundation under static loading was by Tasi and Westmann in 1967 and expanded by Weitsman in 1970 [23,24,25,26,27]. Tensionless foundations experience compressive supporting forces only, which allows for the separation of the rail-tie structure and ballast. These separation points lead to nonlinearity and create mathematical complexity due to the unknown boundaries of the contact regions [28,29]. Zhang et al. have shown that the Winkler and tensionless Winkler foundation models are two special cases of a more general bilinear elastic foundation model, corresponding to the cases of equal stiffness in tension and compression and to zero tensile stiffness, respectively [30]. Various solutions exist in the literature [31,32,33,34,35,36,37,38] for finite and infinite beams resting on tensionless foundations that address various combinations of: uniform [25,31,33] and spatially varying [36,38] foundation stiffnesses and/or gaps [33,34], linear [31,33,36] or nonlinear [25,38] foundation stiffness behavior, varying loading types, and beam boundary conditions. Table 1 summarizes several analytical methods that emphasize one or more of the tensionless foundation attributes. Analytical approaches such as the transfer displacement function method (TDFM) [31] or Green’s function [32], and numerical methods such as Netwon-Ralphson [33], Galerkin [25], or Ritz [35] iteratively identify lift-off points and estimate the deflection profiles of tracks; finite element results [38] indicate the most general modeling capabilities.

In railway applications, both controlled static load tests and moving measurement systems (e.g., instrumented railcars such as MRail) generate deflection data along the track [39,40,41]; however, converting these data streams into accurate and spatially representative track modulus estimates remains a key challenge. For example, MRail-type systems combine lasers and a digital camera mounted on a rail vehicle to measure relative vertical deflection from a moving platform, with GPS and encoder-based localization and a defined calibration procedure that converts image-based measurements into rail-relative deflection [39,42]. These measurements require calibration to convert raw signals into rail-relative deflection profiles, which can then be directly used as inputs to physics-based inverse models. Similarly, controlled static experiments typically employ displacement transducers such as LVDTs and strain gauges to measure deflection basins and load responses, where proper zeroing, load control, and alignment of measurement locations along the rail allow consistent integration with the proposed model framework [43].

Optimal sensor design and deployment of these existing railway track monitoring systems requires (1) a physics model that captures essential features of the system under observation, and (2) compatibility of integration of the model into short-time, computationally efficient monitoring frameworks. The method detailed in this study generalizes load–track condition–track response relationships (i.e., deflection profiles, lift-off points, and shear and moment diagrams) through singularity functions, superposition, and moment–curvature relationships. Ultimately, the method assesses ballasted rail track by estimating foundation stiffness parameters (i.e., track modulus) along the rail. Furthermore, the generality of the method permits direct application to measurement data streams obtained from general ballasted rail track support conditions, including laboratory static load tests (i.e., finite beam) and field (i.e., infinite beam) monitoring systems, regardless of resemblance to pure Winkler conditions. This ensures a consistent interpretation of measured responses while preserving an accurate physical representation of the rail–foundation interaction. These features highlight the method’s potential for practical implementation in evaluating deteriorated track segments [40,44,45], enabling more reliable evaluation of soft or mud spot regions and supporting routine assessment and maintenance decision-making in railroad infrastructure contexts. Section 2 presents theoretical derivations of the physics model describing a flexural rail system supported by a tensionless Winkler foundation. Section 3 details a track modulus estimation framework operating on the model described in Section 2. Section 4 presents six illustrative examples that highlight the method’s generality compared with existing methods. Section 5 and Section 6 provide discussion and conclusions.

## 2. General Finite Beam on Tensionless Foundation Model Derivation

**Figure 1:**
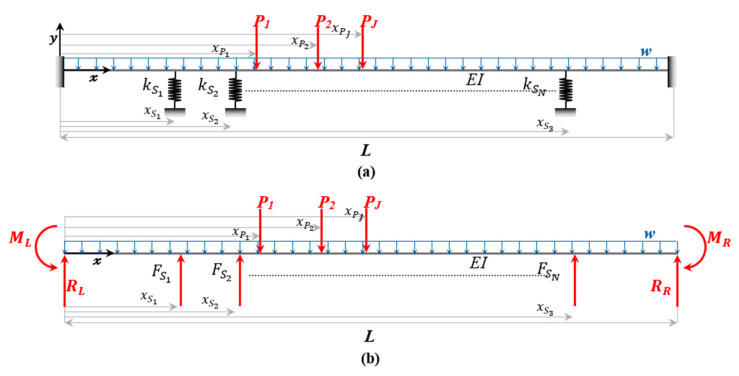
(**a**) Fixed-fixed beam resting on discrete springs. (**b**) Free-body diagram.

Consider a finite, fixed-fixed Euler-Bernoulli beam resting on a series of discrete elastic springs, each with stiffness $k_{S_{n}}$ at distances $x_{S_{n}}$ from the left end of the beam, as shown in Figure 1a. The beam has length *L* and flexural rigidity *EI*. The beam is subjected to a uniformly distributed load, *w*, the self-weight of the rail and ties, and multiple concentrated loads $P_{j}$ at distances $x_{P_{j}}$ from the left end of the beam. While self-weight has a relatively small effect on the overall static deflection in contact regions, it plays an important role in tensionless foundation models by influencing the contact conditions between the rail and the supporting springs.

From Figure 1b, the internal bending moment is obtained by:

(1) $$M\left(x\right)=-M_{L}+R_{L}x-\sum_{j=1}^{J}M_{P_{j}}-M_{W}+\sum_{n=1}^{N}M_{S_{n}}$$

where *x* is the longitudinal coordinate along the beam measured from the left end support. $M_{L}$ and $R_{L}$ are the reaction bending moment and vertical reaction force at the left fixed support, respectively. The indices n=1,2,…,N and *j* = 1, 2, …., *J*. *N* and *J* are the total number of springs and concentrated loads, respectively. $M_{P_{j}}$, $M_{W}$, and $M_{S_{n}}$ are the bending moments due to the concentrated loads, uniform distributed load, and the spring supports, as shown in Equations (2)–(4), respectively.

(2) $$M_{P_{j}}(x)=P_{j}<x-x_{P_{j}}>$$

(3) $$M_{W}(x)=w\frac{x^{2}}{2}$$

(4) $$M_{S_{n}}(x)=F_{S_{n}}<x-x_{S_{n}}>$$

where < > indicate the singularity brackets defined as follows:

(5) $$<x-a{>}^{s}\;={\left(x-a\right)}^{s}\;H\left(x-a\right),\;s\geq 0$$

where *s* is an integer and *H*(·) is the Heaviside function. The spring restoring force is defined as follows:

(6) $$F_{S_{n}}={\left(-1\right)}^{i}k_{S_{n}}\delta_{S_{n}}^{i}$$

where $k_{S_{n}}$ and $\delta_{S_{n}}^{i}$ are the spring constant and the spring displacement raised to the power *i*, capturing the potentially nonlinear effect in the force–displacement relationship, respectively. By utilizing the moment–curvature relationship, as shown in Equation (7).

(7) $$\frac{d^{2}y(x)}{dx^{2}}=\frac{M(x)}{EI}$$

The beam deflection is derived as shown in Equation (8):

(8) $$y(x)=\frac{1}{EI}\left[-\frac{M_{L}x^{2}}{2}+\frac{R_{L}x^{3}}{6}-\frac{\sum_{j=1}^{J}P_{j}<x-x_{P_{j}}{>}^{3}}{6}-w\frac{x^{4}}{24}+\frac{\sum_{n=1}^{N}F_{S_{n}}<x-x_{S_{n}}{>}^{3}}{6}\right]+Cx+D$$

The total number of unknowns in the system is 4 + *N*, with 2 accounting for the integration constants, C and D, 2 accounting for the moment and vertical reactions at the left fixed support, and *N* accounting for the number of displacements at each spring support. To determine these unknowns, the boundary conditions, along with the equations of the displacements at each spring are utilized, as represented in Equations (9) and (10), respectively.

(9) $$y(0)=y(L)=0,\;\;\frac{dy}{dx}(0)=\frac{dy}{dx}(L)=0$$

(10) $$\delta_{S_{m}}=\frac{1}{EI}\left[-\frac{M_{L}x_{S_{m}}^{2}}{2}+\frac{R_{L}x_{S_{m}}^{3}}{6}-\frac{\sum_{j=1}^{J}P_{j}<x_{S_{m}}-x_{P_{j}}{>}^{3}}{6}-w\frac{x_{S_{m}}^{4}}{24}+\frac{\sum_{n=1}^{N}F_{S_{n}}<x_{S_{m}}-x_{S_{n}}{>}^{3}}{6}\right]+Cx_{S_{m}}+D$$

where m=1,2,…,N, and *N* is the total number of springs. After determining the unknowns, the values are then substituted into Equation (8). The beam deflection, y(x), is derived for the system shown in Figure 1a. To solve Equation (8) for other boundary configurations, the common end conditions are summarized in Table 2, where *V(x)* denotes the internal shear force along the beam.

### 2.1. Winkler Foundation

As a benchmark and to approximate the Winkler foundation, discrete springs, *N*, are equally spaced along the length of the contact region, *L_C_*, with spacing:

(11) $$l=\frac{L_{C}}{N}$$

Based on the spacing between the discrete springs, the Winkler foundation stiffness can be approximated using Equation (12).

(12) $$k_{S_{n}}=K\left(x_{S_{n}}\right)l$$

where $K(x_{S_{n}})$ is the Winkler foundation stiffness at $x_{S_{n}}$. This foundation stiffness can vary by varying individual spring stiffnesses along the length of the beam.

### 2.2. Two-Stage Iterative Approach for Tensionless Winkler Foundation

**Figure 2:**
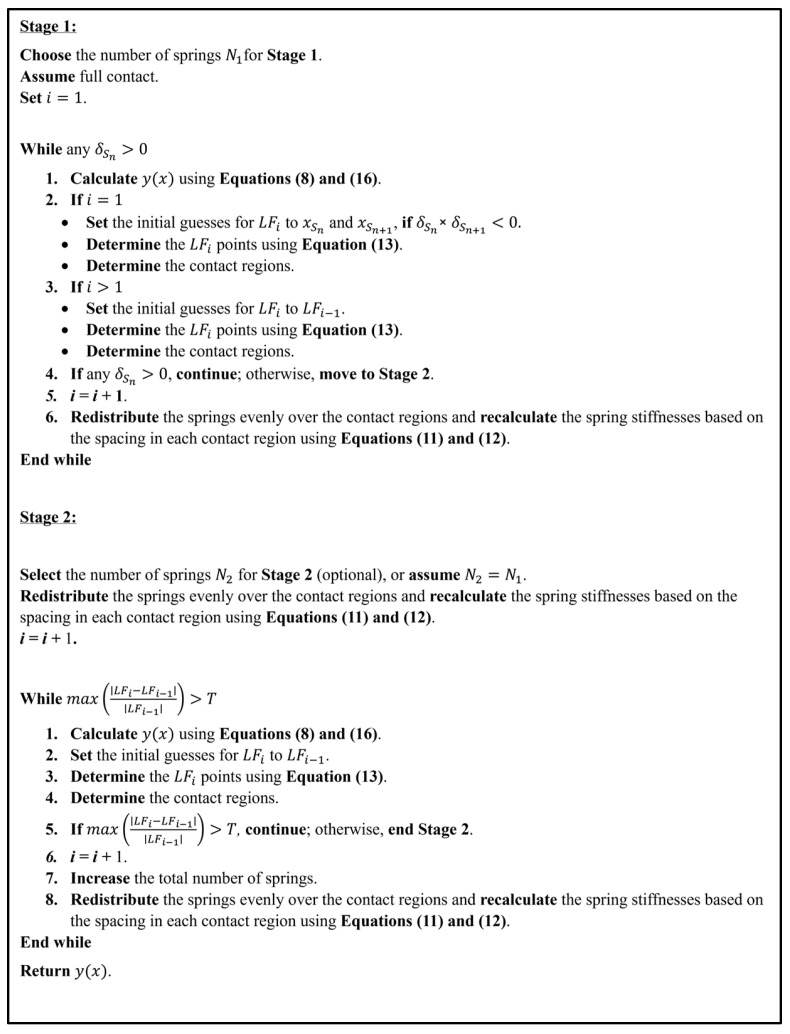
Two-stage iterative approach for beam on tensionless Winkler foundation.

In the method, a two-stage algorithm, as shown in Figure 2, approximates the tensionless Winkler foundation. The spacing between the adjacent springs is initially selected as a fraction of the characteristic length of the track, ensuring that the discretization is physically meaningful and sufficiently fine to capture the deflection profile and contact behavior [46]. Based on this initial spacing, the corresponding number of springs is then determined using Equation (11). In cases involving varying loading conditions or changes in foundation gap and stiffness behavior, a smaller spacing may be adopted. In the first stage, the algorithm identifies the contact and lift-off regions by first assuming that all the springs are connected to the beam and evenly distributed along the beam length. If any of the springs are experiencing tension, it is identified as being in the lift-off region. The algorithm then iteratively redistributes the springs within the contact regions, regions between the lift-off points with y(x)≤0, experiencing compression, and adjusts the springs’ stiffnesses and spacing. At the redistribution step, the algorithm calculates the number of springs for each contact region by rounding up the multiplication of the ratio of contact length to the total contact length times the total number of springs. Therefore, an increment with a minimum of 1 and a maximum equal to the number of contact regions may be added to the total number of springs. The algorithm continues updating until all the springs are in compression. The undeformed profile of the foundation can be represented as a function along the length axis, g(x) for 0<x<L. Therefore, in each iteration, the algorithm determines the locations of the lift-off points by numerically solving for *x* using Equation (13), where y(x) is determined from Equation (8):

(13) y(x)=g(x)

The initial guess, $LF_{0}$, for the lift-off points in the first iteration can be assumed based on the sign of the deflection multiplication of the neighboring springs (e.g., $\delta_{S_{n}}$ and $\delta_{S_{n+1}}$). If the sign is negative, the location of one of these springs is assumed as the initial guess of a lift-off point, including x=0 and L. In the next iteration, the initial guess is the lift-off points from the previous iteration. In the second stage, the algorithm iteratively increases the number of springs and redistributes the springs until a maximum relative change in the lift-off points, $LF_{i}$, satisfies the condition:

(14) $$\varepsilon =\max\left(\frac{\left|LF_{i}\;-\;LF_{i-1}\right|}{\left|LF_{i-1}\right|}\right)\leq T$$

where *LF_i_* and *LF*_*i*−1_ are the lift-off point values at iterations *i* and *i* − 1, respectively. T is the predefined tolerance for convergence. If the condition is satisfied, further iterations are unnecessary, and the solution reaches an optimal accuracy and is considered stable. In a tensionless Winkler foundation, an initial gap $y_{g_{n}\;}$, may exist between the beam and the springs. Therefore, the gap of each spring may be adjusted based on its location, as shown in Equation (15):

(15) $$y_{g_{n}}=g(x_{S_{n}})$$

Therefore, Equation (10) can be rewritten as shown in Equation (16), respectively.

(16) $$\delta_{S_{m}}+y_{g_{m}}=\frac{1}{EI}\left[-\frac{M_{L}x_{S_{m}}^{2}}{2}+\frac{R_{L}x_{S_{m}}^{3}}{6}-\frac{\sum_{j=1}^{J}P_{j}<x_{S_{m}}-x_{P_{j}}{>}^{3}}{6}-w\frac{x_{S_{m}}^{4}}{24}+\frac{\sum_{n=1}^{N}F_{S_{n}}<x_{S_{m}}-x_{S_{n}}{>}^{3}}{6}\right]+Cx_{S_{m}}+D$$

## 3. Application to Track Modulus Characterization

In this section, an iterative static track modulus characterization approach is applied to the model developed in Section 2. A common measure of track support manifests in track modulus which refers to the vertical stiffness of the track foundation and represents the effect of all the structural components beneath the rail (ties, fasteners, ballast, subgrade, etc.) [40,44]. Track modulus is defined as the supporting force per unit length of rail per unit deflection [44,45]. The method developed here essentially fits the model deflection estimates to the observed deflections by adjusting the track modulus.

The calibration algorithm follows a two-stage workflow. First, the measured deflection, rail properties, loading conditions, and an initial discretized foundation are provided as inputs. In Stage 1, the secant method estimates the average track modulus assuming uniform support. The residual between the measured and predicted deflections is then evaluated. If the residual satisfies the convergence criterion, the solution is accepted. Otherwise, Stage 2 is activated, where the Broyden method estimates a spatially varying track modulus by minimizing the norm of the residual of measured and predicted deflections. The final outputs include the track modulus distribution, the identified contact (and lift-off) regions, and the residual error.

The detailed formulation of the two stages is described as follows. The approach utilizes the Secant method in the first stage and the Broyden method (Secant-like) in the second stage to increase numerical efficiency over a grid search or brute force method [47,48]. In the first stage, the algorithm uses the Secant method to solve for the averaged track modulus by considering the track modulus uniform along the rail, as shown in the following expression:

(17) $$K_{i+1}=K_{i}-\frac{f(K_{i})(K_{i}-K_{i-1})}{f(K_{i})-f(K_{i-1})}$$

where *f* is the difference between the observed and estimated deflection under the load (i.e., a single point along the beam). The secant method requires two initial guesses. Therefore, the track modulus initial guesses can be assumed based on the BOEF or Winkler theory, Equation (18) [41], (e.g., *K*_0_ = *K* and *K*_1_ = 1.3*K*).

(18) $$K=\frac{1}{4}\sqrt[{3}]{\frac{P^{4}}{EIw_{m}^{4}}}$$

In this stage, one of two conditions must be satisfied and further iterations cease:

(19) $$\varepsilon_{K}=\left|K_{i}-K_{i-1}\right|\leq T_{K}$$

(20) $$f(K_{i})-f(K_{i-1})=0$$

Method termination based on condition (20) may indicate either convergence of the solution or limitations of the numerical iterative procedure. In some cases, the algorithm may not converge to the global solution for the given model settings. Therefore, such termination is interpreted as an indication that further refinement is required. In the present framework, this refinement is carried out in Stage 2, where the assumption of uniform track modulus can be relaxed to improve the solution. Here, *T_K_* is the predefined tolerance for convergence. The inputs in Stage 1 are the track modulus initial guesses (*K*_0_ and *K*_1_), loads’ magnitudes and locations, rail length and flexural rigidity, deflection profile, and a fixed number of foundation springs, along with initial guesses for lift-off points (i.e., *LF*_0_ assumed as 0 and *L*). The outputs of Stage 1 are the difference between the observed and estimated deflections, represented by the vector *F*, whose components correspond to the residuals at the measurement locations, the average track modulus (*K_avg_*), and lift-off points. If condition (21) is met, then Stage 2 is unnecessary as a single track modulus adequately represents the entire finite beam length.

(21) $$\left\|\vec{F}\right\|\leq T_{F}$$

where *T_F_* is the predefined tolerance for convergence.

On the other hand, if inequality 21 is not satisfied, then a closer match of deflections along the finite beam is achieved by varying the track modulus in a non-uniform manner. In Stage 2, Broyden’s method estimates the varying track modulus. First, the track is discretized into *n* segments of potentially varying track modulus, matching the number of the measurement locations, represented by *n* modification factors, *r*, operating on the *K_avg_* from Stage 1. The response across the segments is coupled through the beam mechanics and contact conditions. In this stage, the method solves a system of nonlinear equations *F(r)* = 0, where *F*: $R^{n}\to R^{n}$, which is equivalent to minimizing the residual norm $\phi (r)={\left\|F(r)\right\|}_{2}\;$. The Broyden method begins with an initial approximate Jacobian matrix and then updates the approximate Jacobian matrix by rank-one corrections, as shown in Equation (25). Therefore, the method will first identify the existence of fixed and free variables in the first few iterations. After determining these variables, the approximate Jacobian matrix reinitiates and updates with each iteration. This is an important step that significantly reduces the total time and number of iterations, as will be discussed in Section 5. The Broyden method requires two initial guesses to initiate the Jacobian matrix and to determine the direction of the line search. Therefore, the first initial guess is by assuming that all the modification factors are equal to 1 and defining upper and lower bounds as 1.5 and 0.5, respectively. These choices stem from the Stage 2 initial track modulus equal to *K_avg_* from Stage 1; the bounds reflect the physical tendency of the rail to mollify the effect of changing track moduli. The method operates on the same inputs as Stage 1, with the modification factors multiplied by the average track modulus, *K_avg_*, comprising a vector input. In Stage 2, the method returns the residual vector *F*_0_ and *LF.* The initial residual norm is $fnorm\;={\left\|F_{0}\right\|}_{2}$. Based on *LF*, any segments located in lift-off regions are assigned a fixed modification factor equal to one. The second initial guess is established by probing in a search direction defined by normalizing the initial residual vector to unit length, which defines the initial probing direction $p_{0}$, and taking a small step, $\tau \;=10^{-2}$, letting “*free*” denote the free index set.

(22) $$r_{probe}=r_{probe}+\tau p_{0}$$

Now, evaluating the function at this probe, which gives a second residual vector *F_probe_*. Next, the initial approximate Jacobian matrix is:

(23) $$B\left(free\right)=\gamma I$$

where *I* is an identity matrix with *n_free_* × *n_free_* size, and γ is the approximate directional derivative calculated as follows:

(24) $$\gamma =\frac{{p{\left(free\right)}^{T}}_{0}\left[{F(free)}_{probe}-F_{0}(free)\right]}{\tau }$$

After initiating the approximate Jacobian matrix, the method starts the main Broyden iteration loop. In this loop, the method repeats until convergence, Equation (21). Each iteration *i* of the loop performs the following steps:

1. Solve for a search direction, *p*, by solving Equation (25):

(25) $$B{\left(free\right)}_{j-1}\;{p(free)}_{j}=-F{\left(free\right)}_{j-1}$$

2. Line search and bounds:

Compute *α_j_*, so that $r_{j}\;+\;\alpha_{j}p_{j}$ stays within the bounds (*l* and *u*).

Where *α_j_* is:

(26) $$\alpha_{j}=min\left\{\begin{array}{c} \underset{i\in M}{\min}\frac{u_{i}-{r(free)}_{i}}{{p(free)}_{i}},\mathrm{M are the indices where}\;p_{i}>0\\ \underset{i\in Q}{\min}\frac{l_{i}-{r(free)}_{i}}{{p(free)}_{i}},\mathrm{Q are the indices where}\;p_{i}<0\\ 1 \end{array}\right.$$

Then, check whether *p*(free) is a descent direction for the objective function.

The vector p(free) is a descent direction, since

(27) $${\left[B(free)p(free)\right]}^{T}F(free)\;<\;0$$

If the condition is not satisfied, p(free)=−p(free).

3. Free and fixed variables adjustment and trial step (inner loop).
  The process starts by adjusting the free and fixed variables before taking a trial step within the inner loop. Once this adjustment is completed, a new trial step is calculated using $r_{Try}=r+\alpha_{j}p_{j}$. The method then compares the newly identified free variables with those determined from the previous iteration or trial. If the free variables remain unchanged, the inner loop terminates. However, if the free variables differ, the variables are adjusted again and the inner loop continues until the set of free variables stabilizes.

4. Accept the step.

$$r=r_{Try}\;\mathrm{and}\;F=F_{Try}$$

5. Jacobian matrix (Broyden) update.
  If the free variables set remains unchanged, the Jacobian matrix is updated using Broyden rank-1 correction, as shown in Equation (28).
  (28) $${B(free)}_{j+1}={B(free)}_{j}+\frac{\left(y_{j}-{B\left(free\right)}_{j}s_{j}\right)s_{j}^{T}}{s_{j}^{T}s_{j}}$$
  where $y_{j}={F(free)}_{j+1}-{F(free)}_{j}$ and $s_{j}={r(free)}_{j+1}-{r(free)}_{j}$. However, if the set of free variables changes, the Jacobian matrix is reinitialized instead of updated, as shown in Equation (29).
  (29) $${B(free)}_{j+1}=\frac{s_{j}^{T}y_{j}}{s_{j}^{T}s_{j}}I$$

6. Check the convergence.
  Convergence is then checked using Equation (25); if the convergence is satisfied, the method ends and the overall output is the track modulus as a function of the deflected length.

**Figure 3:**
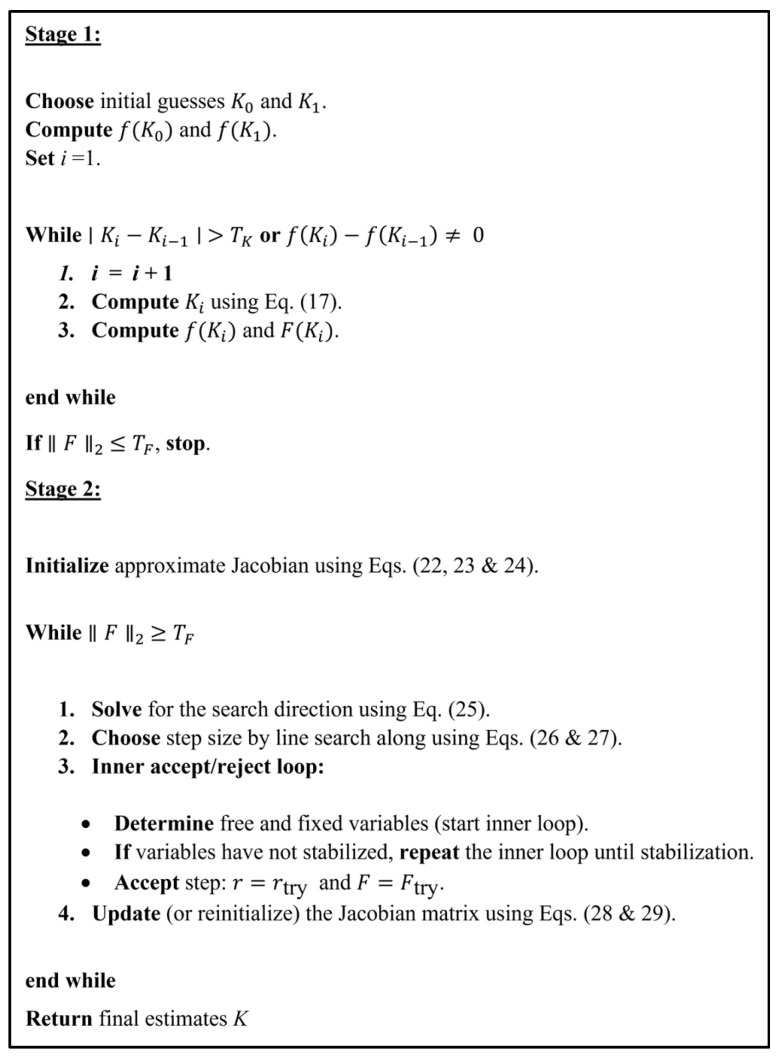
Two-stage iterative procedure for track modulus estimation: Stage 1 employs the secant method, while Stage 2 uses the Broyden method.

A summary of the proposed approach is shown in Figure 3.

## 4. Numerical Examples

This section demonstrates the robustness and effectiveness of the proposed model through six illustrative examples. The proposed method is validated by comparing its prediction against several solutions selected from the literature and finite element models, as summarized in Table 1. These examples cover a variety of beam–foundation configurations and assumptions, including both infinite and finite beams, uniform and spatially varying foundation stiffness or gap, linear or nonlinear foundation stiffness behavior, loading types, and beam boundary conditions. In addition to the validation examples, one illustrative numerical example is presented to demonstrate the potential of the proposed method for track modulus characterization. The track modulus characterization approach is shown in Figure 3. This example demonstrates how the proposed method can be adapted for practical track evaluation for either laboratory or field data. Overall, these validations illustrate the accuracy, reliability, and general applicability of the proposed model for analyzing beam–foundation interactions under varied conditions and the potential applicability of the proposed model for static track modulus characterization.

### 4.1. Example 1: Infinite Beam Resting on a Tensionless Foundation with a Gap at the Interface

**Figure 4:**
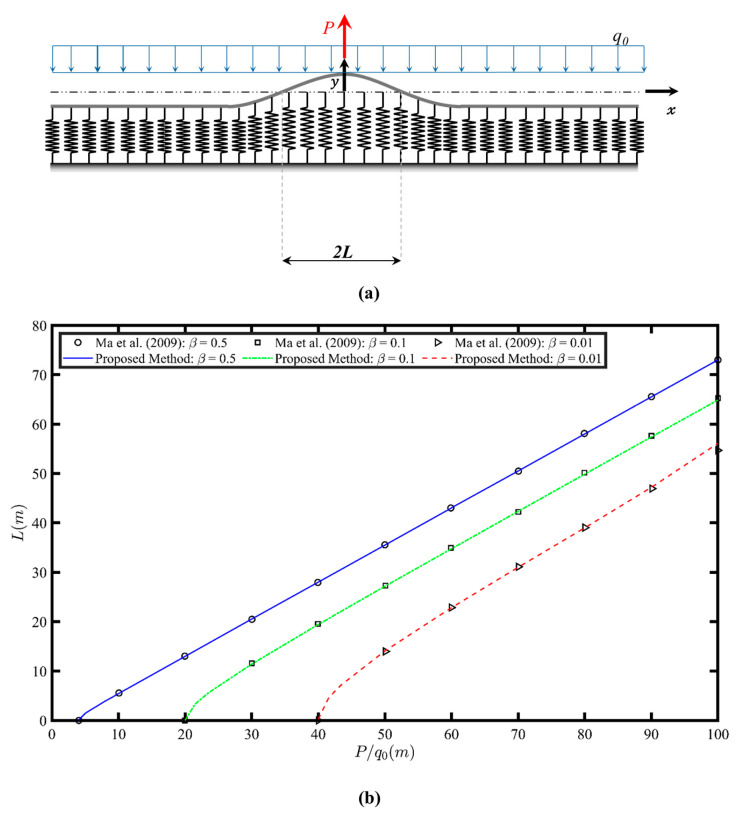
(**a**) Infinite beam resting on tensionless Winkler foundation and subjected to its self-weight and a central upward point load, adapted from Ma et al. (2009) [31]. (**b**) Central non-contact region length L as a function of the P/q_0_ ratio. The results from Ma et al. (2009) are included for comparison [31].

In this example, two cases are considered and compared to the solutions provided by Ma et al. [31]. In the first case, an infinite beam subjected to its self-weight, $q_{0}$ (where $q_{0}=\;\rho gA$), resting on a tensionless foundation, as shown in Figure 4a. An upward point load *P* is applied at the beam and the origin of the coordinate system is centered at this load due to the system’s symmetry. The point load induces separation between the beam and the foundation, resulting in a lift-off region at the center. The length of the lift-off region is determined. Since the proposed method is applied to a finite beam, the length of the finite beam is taken to be long enough, such that the deflection becomes independent of the length and the effect of the boundary is considered negligible. The utilized finite beam has free-free boundary conditions and the origin of the *x-axis* was shifted to the point load location. As shown in Figure 4b, the lift-off length of the beam is plotted against the ratio of $P/q_{0}$ for different values of the foundation stiffness parameter $\beta =\sqrt[{4}]{\frac{K}{4EI}}$. The results from the proposed method align with the graphical solution provided by Ma et al. [31].

**Figure 5:**
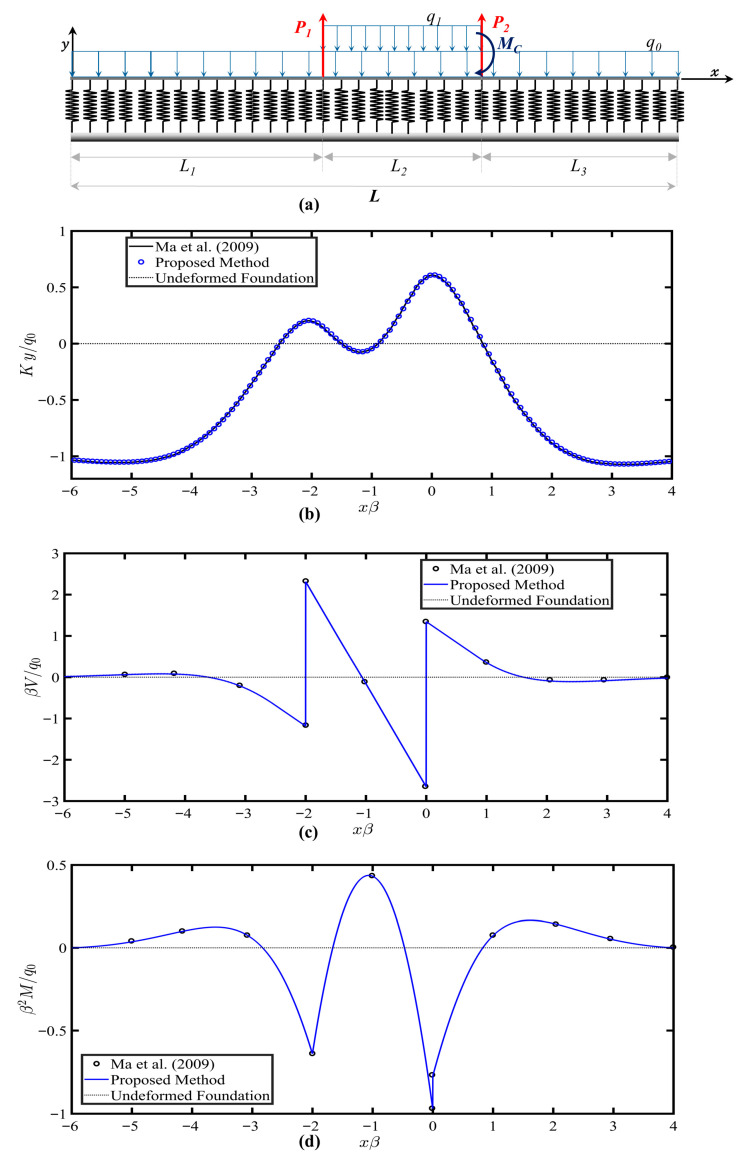
(**a**) Infinite beam resting on tensionless Winkler foundation and subjected to arbitrary loads. (**b**) Beams’ deflection. (**c**) Shear force diagrams. (**d**) Bending moment diagram. The results from Ma et al. (2009) are included for comparison [31].

In the second case, the infinite beam is subjected to arbitrary loads, as shown in Figure 5a. Table 3 shows the beam and foundation parameters. The results for free-free beam displacement, shear force and bending moment responses are shown and compared with the infinite beam responses provided by Ma et al. [31] in Figure 5b–d, respectively. Moments along the length axis due to the concentrated moment, *M_C_*, and distributed load, *q_1_*, can be derived as shown in Equations (30) and (31), respectively:

(30) $$M_{M_{C}}\left(x\right)=M_{C}<x-\left(L_{1}+L_{2}\right){>}^{0}\;$$

(31) $$M_{q_{1}}=\frac{q_{1}}{2}\left[<x-L_{1}{>}^{2}-<x-\left(L_{1}+L_{2}\right){>}^{2}\right]$$

For consistency with the infinite beam and the source formulation, the origin of the *x*-axis is shifted to $x_{p_{2}}$ and only results for −60 m≤x≤40 m are shown. Results are presented in dimensionless form. Results showed an excellent agreement with the solution provided graphically by Ma et al. [31], confirming the capability of the method in estimating the static responses of infinite beams.

### 4.2. Example 2: Finite Beam Resting on a Uniform Tensionless Foundation with a Uniform Gap at the Interface

**Figure 6:**
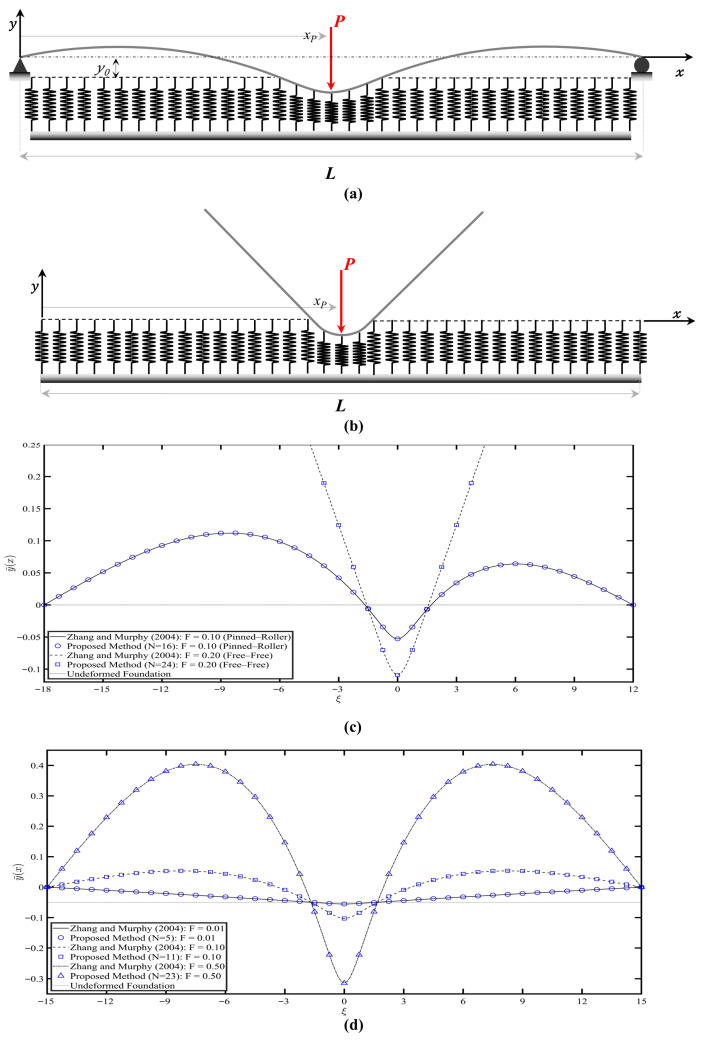
(**a**) Simply supported beam resting on tensionless Winkler foundation with a gap *y*_0_ and subjected to a point load applied at *xp*. (**b**) Free-free beam resting on tensionless Winkler foundation and subjected to a point load applied at *xp*. (**c**) Deflections of simply supported and free-free beams under a point load at $x_{p}=0.6\bar{L}$, with zero gap ${\bar{y}}_{0}=0$. (**d**) Deflections of a simply supported beam under a point load at $x_{p}=0.5\bar{L}$, with a non-zero gap ${\bar{y}}_{0}=0.05$. The results from Zhang and Murphy (2004) are included for comparison [33].

Two different boundary conditions are utilized in this comparison: pinned-roller and free-free, as shown in Figure 6a,b, respectively. The beam has length L and is resting on a tensionless Winkler foundation and separated by a gap, $y_{0}$. In Zhang and Murphy [33], the nondimensional governing equation is derived with the coordinate origin at $x_{p}$. For consistency with the present formulation, the origin of the x-axis is shifted to $x_{p}$ (by defining $\hat{x}=x-x_{p}$, as the new horizontal coordinate) and normalized with respect to the foundation stiffness parameter β. The nondimensional governing equation obtained by Zhang and Murphy [33]:

(32) $$\frac{d^{4}\bar{y}}{d\xi^{4}}=0,\;\;\;\xi \in \left[-{\bar{x}}_{p},-{\bar{L}}_{c1}\right]\cup \left[{\bar{L}}_{c2},\bar{L}-{\bar{x}}_{p}\right]$$

(33) $$\frac{1}{4}\frac{d^{4}\bar{y}}{d\xi^{4}}+\bar{y}-{\bar{y}}_{0}=F\delta \left(\xi \right),\;\;\xi \in \left[-{\bar{L}}_{c1},{\bar{L}}_{c2}\right]$$

where the nondimensional parameters are given by:

(34) $$\bar{y}=\beta y,\;\;{\bar{y}}_{0}=\beta y_{0},\;\;\xi =\beta \hat{x},\;\;{\bar{x}}_{p}=\beta x_{p}$$

(35) $${\bar{L}}_{c1}=\beta L_{c1},\;\;{\bar{L}}_{c2}=\beta L_{c2},\;\;\bar{L}=\beta L,\;\;F=\frac{P}{4\beta^{2}EI}$$

The nondimensional displacement, $\bar{y}(x)$, is plotted against the nondimensional position, *ξ*, as shown in Figure 6c. The simply supported beam is subjected to a dimensionless force F = 0.1, while the free-free beam is subjected to a dimensionless force F = 0.2. A nondimensional beam length $\bar{L}\;=\;30$ has been considered for both beams in the analysis, with the applied load at $x_{p}=0.6\bar{L}$, and a gap ${\bar{y}}_{0}=0$. An excellent agreement, quantified by a mean squared error (MSE) of 6.8 × 10^−7^ and 1.14 × 10^−6^ for simply supported and free beams, respectively, between the proposed method and the analytical solution, confirms the capability of the proposed method to model the system with precision, achieved with a tolerance set to $\varepsilon =10^{-5}$. The results show that the pinned-roller case achieved convergence with 16 springs, whereas the free-free beam required 24 springs to reach convergence. However, when the gap size is nonzero, only the pinned-roller beam is considered. Therefore, to verify the method’s reliability, consider a ${\bar{y}}_{0}=0.05$, $x_{p}=0.5\bar{L}$ for different values of the dimensionless force F=0.01, F=0.1 and F=0.5, using the convergence tolerance of $\varepsilon =10^{-3}$, $\varepsilon =10^{-4}$, and $\varepsilon =10^{-5}$, respectively. Tighter tolerances were adopted for higher loads to maintain computational stability and accuracy. As the load increased, the contact region of the beam penetrated deeper into the foundation, resulting in increased distance between the adjacent spring contact nodes, despite the uniform horizontal spacing. As shown and compared in Figure 6d, the agreement between the proposed method and the analytical method is excellent for all applied load levels considered: MSE of 5.35 × 10^−8^, 2.51 × 10^−8^, and 3.4 × 10^−7^ for F values of 0.01, 0.1, and 0.5, respectively. Overall, the results demonstrate the proposed method’s effectiveness and reliability, and higher accuracy can be achieved when lower tolerance thresholds are applied.

### 4.3. Example 3: Finite Beam Under Sinusoidal Loading on a Tensionless Foundation

**Figure 7:**
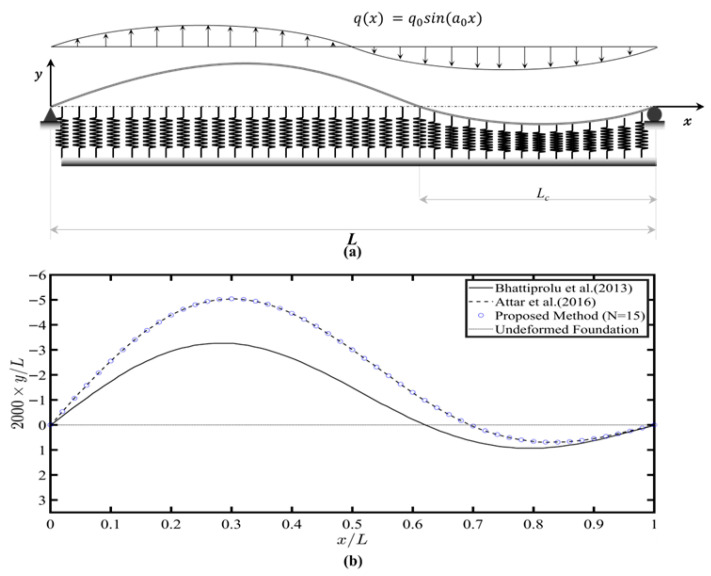
(**a**) Pinned-roller beam resting on tensionless Winkler foundation and subjected to antisymmetric sinusoidal load. (**b**) Beam deflection under an antisymmetric sinusoidal load. The results from Bhattiprolu et al. (2013) are included for comparison [25].

To further evaluate the proposed method’s performance, a simply supported beam resting on a tensionless Winkler foundation and is subjected to an antisymmetric sinusoidal load, as shown in Figure 7a. The beam is subjected to an antisymmetric sinusoidal load, $q(x)\;=q_{0}sin(a_{0}x)$, with $q_{0}=-\frac{40}{\pi }\;Nm^{-1}$ and $a_{0}=\frac{2\pi }{L}$. The beam has a length L = 300 mm, cross-sectional area A=1 mm×25.4 mm, and a young’s modulus E=70 GPa. The analysis adopts foundation stiffness K =42.5 kPa. The results using the proposed method are compared against solutions previously obtained by Bhattiprolu et al. [25] using a multimodal approach and by Attar et al. [36] using the discrete lattice spring model (LSM). As illustrated in Figure 7b, the results obtained by the proposed method precisely match the solution provided by Attar et al. [36]. The noticeable underestimation by Bhattiprolu et al.’s [25] solution is attributed to an insufficient number of utilized modes, as previously highlighted by Attar et al. [36]. In contrast to the modal method, the proposed method addressed the tensionless foundation using only 15 springs and a convergence tolerance of $10^{-5}$.

### 4.4. Example 4: Finite Beam Resting on a Non-Uniform Tensionless Foundation

**Figure 8:**
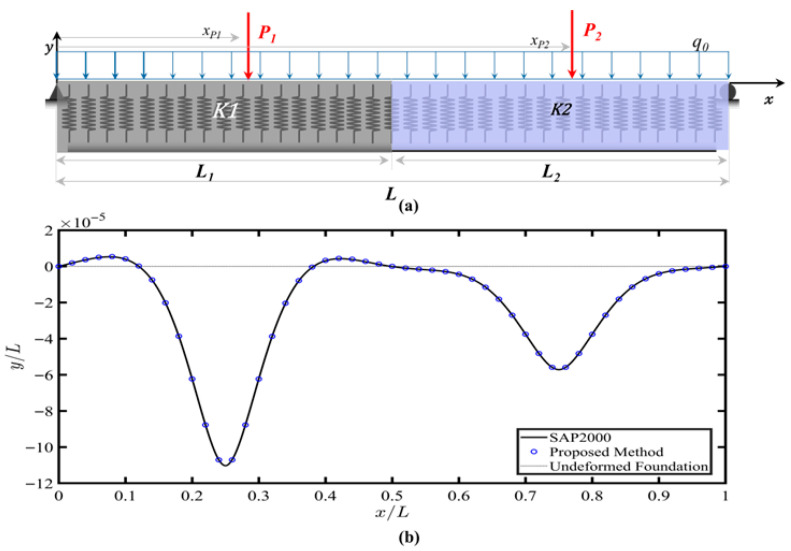
(**a**) Simply supported beam resting on tensionless Winkler foundation with varying foundation stiffness and subjected to two-point loads and a uniform distributed load. (**b**) Deflection profile. The finite element model results are included for comparison.

In this example, a non-uniform track support was evaluated: a simply supported, resting on a tensionless Winkler foundation with varying foundation stiffness and subjected to two-point loads and a uniform distributed load, as shown in Figure 8a. Table 4 shows the beam and foundation parameters. Excellent agreement, MSE of 4.05 × 10^−9^, between the proposed method and FE results, as shown in Figure 8b, demonstrated the capability of the proposed method in addressing finite beams on non-uniform foundations, a critical feature of foundation monitoring.

### 4.5. Example 5: Finite Beam Resting on a Tensionless Foundation with Nonlinear Stiffness Behavior and a Variable Gap

**Figure 9:**
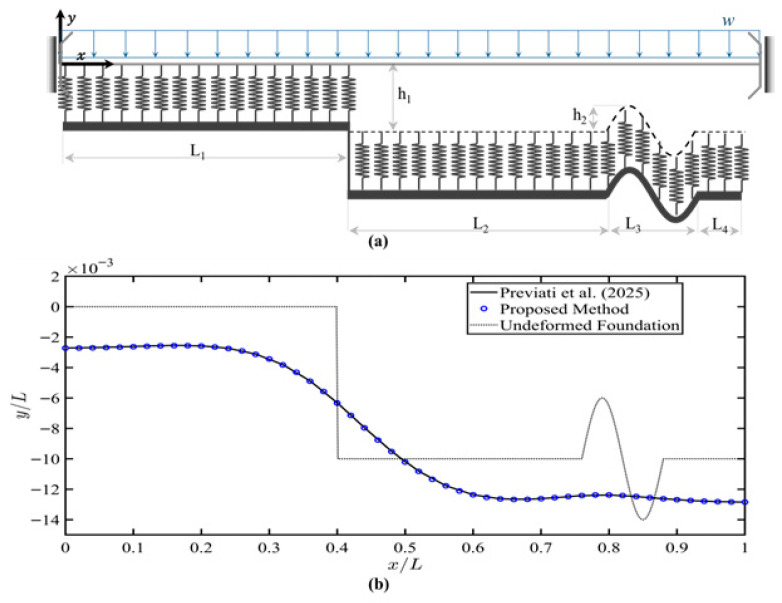
(**a**) Slider-slider beam resting on tensionless Winkler foundation with nonlinear stiffness behavior and a variable gap. (**b**) Beam deflection under the beam’s self-weight load. The results from Previati et al. (2025) are included for comparison [38].

This example investigated a slider-slider beam resting on a nonlinear tensionless Winkler foundation with a nonuniform gap profile, using the same configuration as in Previati et al. [38], as shown in Figure 9a. Previati et al. intend this configuration as a simplified model of a pipe on a terrain with a hollow cross-section uniformly loaded by its self-weight. While not a railway track, the mechanics vary only by the general shape of the cross-section and the applicability of the method was readily assessed. The foundation profile was irregular and varied along the beam length. As shown in Figure 9a, the beam had zero gap from the left support up to 20 m, while from 20 m to 38 m and 44 m to 50 m, a gap of 0.5 m between the beam and the foundation. A sinusoidal shape gap was considered between 38 m and 44 m away from the left support. Table 5 shows the beam and foundation parameters. The foundation stiffness was nonlinear and described by the following expression:

(36) $$K=nk_{0}{∆}_{y}^{n-1}$$

which represented the relationship between the sinkage (Δ*_y_*) and terrain stiffness [38,49]. The sinkage Δ*_y_* represented the deformation of the foundation. Variables *k*_0_ and *n* described the properties of the terrain and accounted for the terrain dimensions. A nonlinear solver (fsolve in MATLAB R2024b) was implemented to solve the nonlinear system of equations. As shown in Figure 9b, the numerical results showed an excellent agreement with FE results provided graphically by Previati et al. [38]. Overall, the proposed model provided an accurate and reliable representation of the nonlinear foundation with a varying gap profile, confirming its effectiveness for simulating complex beam–foundation interaction.

### 4.6. Example 6: Numerical Illustration of the Proposed Method’s Applicability for Track Modulus Characterization

**Figure 10:**
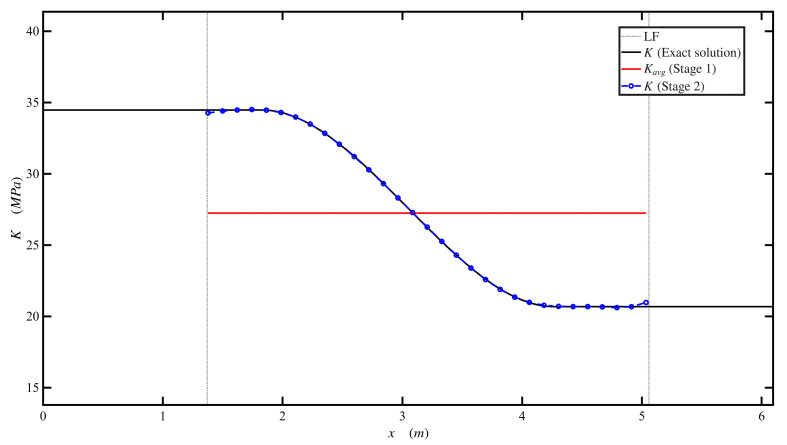
Track modulus estimation.

**Figure 11:**
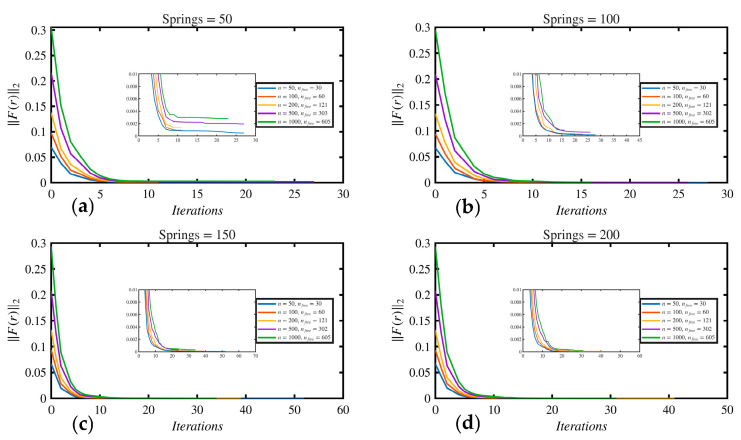
Convergence of the inverse solver for different measurement resolutions (*n*): (**a**) 50 springs, (**b**) 100 springs, (**c**) 150 springs, (**d**) 200 springs.

**Figure 12:**
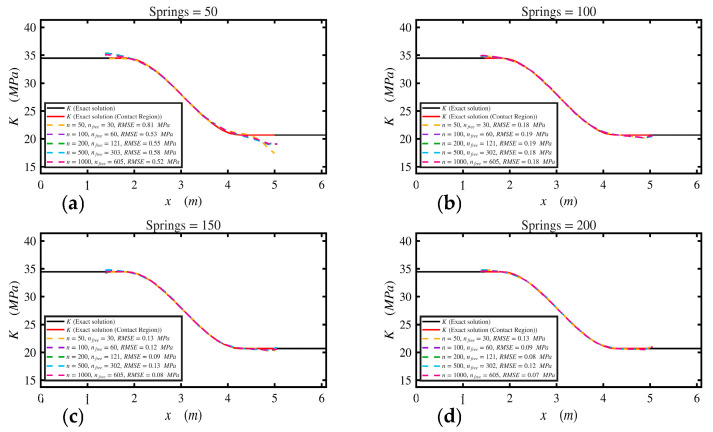
Recovered track modulus for different measurement resolutions (*n*): (**a**) 50 springs, (**b**) 100 springs, (**c**) 150 springs, (**d**) 200 springs. Some colors may not be visually distinguishable because the proposed-method curves nearly overlap.

In this example, the track modulus varied spatially, $3.45\;\times 10^{7}\frac{N}{m^{2}}$ for $0\leq x\leq \frac{3L}{10}\;$ and then gradually decreased to $2.07\times 10^{7}\frac{N}{m^{2}}$ for $\frac{7L}{10}\leq x\leq L$, as shown in Figure 10. The point load was applied at midspan. Table 6 shows the beam and foundation parameters. The proposed approach using the Stage 1 (Secant method) estimated the average track modulus, *K_avg_*, as $2.675\times 10^{7}\frac{N}{m^{2}}$. In Stage 2 (i.e., Broyden method), the proposed approach closely estimated the varying track modulus; both Stage 1 and Stage 2 results are shown in Figure 10. A total of 1000 segments were used to discretize the track modulus regions along the beam, and 605 segments were within the contact region, with free variables between 1.37 m and 5.06 m. Upper and lower bounds are set to be 1.5 and 0.5, respectively. Therefore, *n_free_* were 605 within the analysis. The predefined tolerance for convergence was $1\times 10^{-5}$. The results reached a convergent solution at 25 iterations, and the corresponding norm was $8\times 10^{-6}$. Results at the lift-off region (e.g., fixed variables between 0 m and 1.37 m and 5.06 m to 6.096 m) are neglected and not considered since they do not impact the deflection of the rail. A sensitivity study was performed to assess the effect of both measurement resolution (*n*) and foundation discretization on the inverse solution, as shown in Figure 11 and Figure 12. The convergence histories in Figure 11 indicate that all cases exhibit a rapid reduction in the residual norm during the initial iterations. However, for 50 springs, the residual stabilized at higher levels, reflecting limited model resolution. Increasing the number of springs to 100 significantly improved convergence, with faster decay and lower final residual across all values of *n*. For 150 and 200 springs, the convergence curves nearly overlap, indicating stable and consistent behavior independent of measurement resolution.

The corresponding modulus distributions in Figure 12 show that the 50-spring case produces noticeable deviation from the exact solution, particularly near the boundaries of the contact region. In contrast, using 100 springs led to a significant improvement, with the recovered profiles closely matching the exact solution for all *n* values. Further increases to 150 and 200 springs provided a minor improvement, with RMSE values falling below 0.1 MPa and minimal differences between solutions. Overall, these results confirm that the accuracy and convergence of the inverse solution are governed primarily by the foundation discretization, and once a sufficient number of springs is used, the solution becomes robust and largely independent of the number of measurement points.

**Figure 13:**
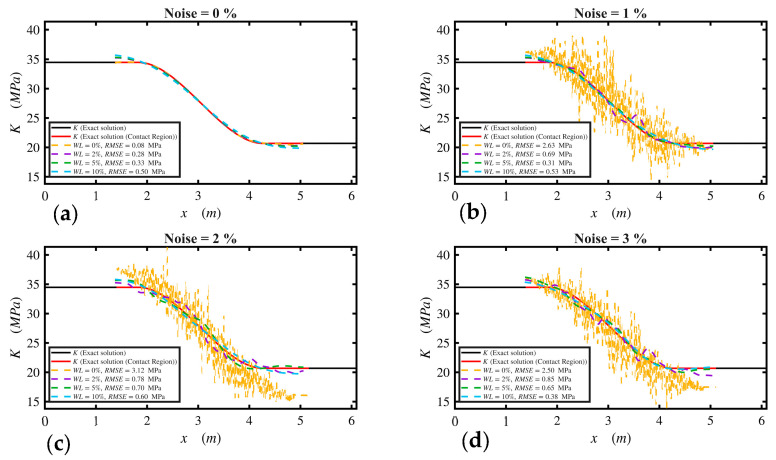
Effect of measurement noise and smoothing window length on the estimated track modulus *K(x)*: (**a**) 0% noise, (**b**) 1% noise, (**c**) 2% noise, (**d**) 3% noise. Some colors may not be visually distinguishable because the proposed-method curves nearly overlap.

Additionally, a noise sensitivity analysis was conducted to evaluate the stability of the inverse solution. Figure 13 illustrates the effect of moving-average window length, WL, on the estimated track modulus *K*(*x*) under increasing noise levels of 0%, 1%, 2% and 3%. A moving average was applied to both the residual vector *F* and the modification factors *r* in Stage 2, with the window length defined as a percentage of the total number of measurements along the track.

The results show that, in the absence of smoothing (WL = 0%), the recovered modulus exhibits significant oscillations under noisy conditions. Introducing a small window (WL = 2%) reduced these fluctuations but still retained a noticeable noise-induced variability. Increasing the window length to 5% effectively suppressed oscillations while preserving the overall spatial trend of *K*(*x*), resulting in improved agreement with the exact solution across all noise levels. A larger window (WL = 10%) produced a smoother response but suppressed local features of the solution.

Across all noise levels, the results indicate that moderate smoothing is sufficient to control noise-induced oscillations without significantly altering the underlying distribution. These findings highlight that the proposed approach remains stable under noisy conditions, provided that an appropriate level of smoothing is applied.

## 5. Discussion

The results demonstrated that the proposed method provides an accurate and stable approach for analyzing beams resting on a tensionless elastic foundation. In all the examples, the method correctly identifies the contact/non-contact regions by allowing separation where the beam deflected away from the foundation. This behavior reflects the physical response of a finite beam resting on a tensionless elastic foundation and confirms that the proposed method enforces the tensionless constraint. The agreement with analytical and finite elements solutions confirmed the accuracy of the proposed method for both finite and infinite beam cases.

The method also showed reliable performance under different loading conditions, including point loads, distributed loads, and sinusoidal loading. In addition, the method captured the effect of uniform and non-uniform foundation stiffness, gaps at the interface, and linear and nonlinear foundation behavior. These results confirm the generality and applicability of the proposed method to represent realistic beam–foundation interaction.

In Example 1, the method accurately predicted the lift-off region length and the beam responses under arbitrary loading. The agreement with the solution provided by Ma et al. [31] confirmed that using a sufficiently long finite beam can represent infinite beam behavior without significant boundary influence. Example 2 demonstrated the method’s ability to analyze finite beams with different boundary conditions and uniform interface gaps. The observed increase in the contact region depth with increasing load was consistent with the expected structural behavior, as the higher loads caused greater beam penetration into the foundation.

In Example 3, the method successfully captured the response of a beam subjected to a sinusoidal loading, which illustrated that the method can effectively handle the varying, non-uniform distributed load and accurately capture the contact/non-contact regions. The comparison also showed improved accuracy compared to the multimodal approach. Example 4 showed the ability of the method to analyze beams resting on non-uniform foundation stiffness. The results confirmed that the method was capable of representing spatial variation in the foundation stiffness. This capability is important for practical applications, as real track support conditions often vary along the length due to differences in material properties, support conditions, or degradation.

Example 5 extended the analysis to nonlinear foundation behavior with a variable gap profile. The method remained stable and accurately predicted beam deflection under nonlinear stiffness conditions. This illustrated the ability of the proposed method to handle realistic foundation conditions where the foundation stiffness depends on the magnitude of the foundation deformation.

Example 6 showed the applicability of the proposed method for track modulus characterization. The results showed that the average track modulus could be estimated using the Secant method, while the spatial variation was accurately captured using the Broyden update procedure. The method successfully estimated the varying track modulus within the contact region. The two-stage approach improved the efficiency and stability of the solution. Stage 1 provided an estimate for average track modulus, *K_avg_*, which would reduce the total number of iterations in the case that the foundation was uniform or nearly uniform and avoid unnecessary iterations. On the other hand, this step is important for Stage 2 because it provides a reasonable reason to use upper, 1.5, and lower, 0.5, bounds, which improved convergence stability and ensured that the modification factors remained within physically meaningful limits.

The sensitivity study further showed that the accuracy of the inverse solution is primarily governed by the foundation discretization (i.e., no. of springs) rather than the number of measurements along the rail. Increasing the number of springs improved the recovered modulus and reduced error, while beyond a certain discretization level, the solution became stable and largely independent of the number of measurement points. Furthermore, the noise analysis showed that the method remains robust under realistic measurement noise, provided that moderate smoothing is applied. Without smoothing, noise introduced oscillation in the recovered modulus, whereas an appropriate window length effectively stabilizes the solution without compromising the underlying spatial variation.

## 6. Conclusions

This paper has presented an analytical method to model the static response of finite beams resting on a tensionless Winkler foundation. The model improves generality by capturing the tensionless nature of beam–foundation interaction based on a two-stage algorithm and without relying on prior knowledge about the contact region boundaries. Specifically, the foundation is modeled as discrete, independent linear springs. In the first stage, the contact regions are identified via an iterative procedure. In the second stage, the springs are redistributed within the contact regions only, and the algorithm iteratively increases the number of springs until it reaches a convergent solution. The iterative solution strategy provides stable convergence and captures the nonlinear mechanics of the tensionless interactions under various loading scenarios. Unlike the Winkler foundation, which assumes continuous contact and an unrealistic identical reaction for both tension and compression, the proposed model restricts the supports to react only in compression. The model not only simplifies the implementation but also shows the applicability to cases involving nonlinear stiffness behavior and spatially varying gap and foundation stiffness, either in a piecewise or continuous manner. The performance of the model has been shown and compared to several examples. In all cases the model converged closely—either graphically or quantitatively if data was available to the target solutions—confirming its accuracy, efficiency, and general applicability.

The model also provides a practical tool for tracking modulus estimation, improving defect detection, and supporting maintenance decisions within railroad infrastructure management. The method allows spatial variation in the track stiffness to be quantified and enables the detection of localized support degradation. Unlike many existing methods, the proposed method is formulated to accommodate both finite rail models, such as laboratory-scale setups, since it allows for the effect of the boundary conditions, and infinite rail models under static loads. Overall, these features enhance the assessment of the track conditions under static load conditions.

In addition, the proposed framework improves the interpretation of measurement data for railway track monitoring by providing a consistent, physics-based interpretation of the measured deflection data. Static load tests typically provide measurements at specific locations, while moving measurement systems (e.g., MRail system) generate continuous deflection profiles along the track. Although the present study focuses on static responses, the formulation can be used to interpret the deflection obtained from different sensing approaches. In particular, the framework can be incorporated into moving measurements, when the measured response can be approximated as quasi-static.

In practice, the calibration of foundation parameters is guided by the measurement process. In controlled laboratory conditions, gap profiles may be known or estimated based on the test setup and boundary conditions. In contrast, the stiffness behavior and contact conditions can be inferred from the measured load-deflection response. Indeed, the model provides a systematic approach to data stream and sensor design by establishing signal-to-noise ratios, measurement locations, and identifying critical quantities requiring further a priori estimation, i.e., gap profiles, etc.

The proposed method has been discussed for static responses; further development involves extending the method into dynamic responses. In practical applications, beams and railway tracks are subjected to moving and time-dependent loads, which introduce inertia and damping effects that influence the structural responses and contact behavior. Extending the method into dynamic responses will enhance the capability of the method to analyze beam–foundation interaction under realistic loading conditions and improve track modulus characterization in dynamic environments.

## Figures and Tables

**Table 1:** Comparative summary of beam models on tensionless foundation as reported by different researchers.

Attributes	Ma et al. (2009) [31]	Zhang & Murphy (2004) [33]	Bhattiprolu et al. (2013) [25]	Attar et al. (2016) [36]	Previati et al. (2025) [38]	Proposed Method
Solution	Numerical-analytical (TDFM)	Analytical/Numerical (Newton Raphson)	Analytical-numerical (Galerkin + Newton Raphson)	Numerical (Lattice spring model)	Numerical (Finite Element/variable reduction)	Approximate analytical
Foundation Type	Tensionless Winkler	Tensionless Winkler	Bilateral & unilateral foundations	Tensionless Winkler	General	Tensionless Winkler
Stiffness Behavior	Linear	Linear	Linear & nonlinear	Linear	Linear and nonlinear	Linear and nonlinear
Stiffness Variation	Uniform	Uniform	Uniform	Variable (piecewise)	Variable (piecewise and continuous)	Variable (piecewise and continuous)
Gap Variation	Uniform	Uniform	Uniform	Uniform	Variable (piecewise and continuous)	Variable (piecewise and continuous)
Loads	Arbitrary	Single concentrated load	Arbitrary	Arbitrary	Arbitrary	Arbitrary
Boundary Conditions	Infinite beam	Free–free and simply supported	Simply supported	General	General	General
Contact Region	Multiple	One	Multiple	Multiple	Multiple	Multiple

**Table 2:** Boundary conditions for different types of end constraints.

Type of End Constraint at *x* = 0 and *x* = *L*	Boundary Conditions
Pinned-roller	y(0)=y(L)=0 M(L)=0
Fixed-free	$y(0)=\frac{dy(0)}{dx}=0$ M(L)=0 V(L)=0
Free-free *	M(L)=0 V(L)=0
Slider-slider *	$\frac{dy(0)}{dx}=\frac{dy(L)}{dx}=0$ V(L)=0
Fixed-pinned	$y\left(0\right)=y\left(L\right)=0$ $\frac{dy(0)}{dx}=0$ M(L)=0

* *N* ≥ 2 to ensure the stability of the system.

**Table 3:** Beam and foundation parameters for Example 1, Case 2.

Parameter	Value
*L*	150 m
*L*_1_	65 m
*L*_2_	20 m
*L*_3_	65 m
Flexural rigidity (*EI*)	${2.5\times 10}^{8}\;Nm^{2}$
Winkler foundation stiffness (*K*)	$10^{5}\;N/m^{2}$
*P*_1_	35,000 N
*P*_2_	40,000 N
*q*_0_	1000 N/m
*q*_1_	1500 N/m
*M_C_*	20,000 Nm

**Table 4:** Beam and foundation parameters for Example 4.

Parameter	Value
*L*	18.29 m
*L*1	9.14 m
*L*2	9.14 m
$x_{p1}$	4.57 m
$x_{p2}$	13.72 m
Flexural rigidity (*EI*)	$8.18\times 10^{6}\;Nm^{2}$
*K*1	$3.45\times 10^{7}\;N/m^{2}$
*K*2	$2.07\times 10^{7}\;N/m^{2}$
*P*1	133.4 kN
*P*2	44.48 kN
*q*_0_	1.75 kN/m

**Table 5:** Beam and foundation parameters for Example 5.

Parameter	Value
*L*1	20 m
*L*2	18 m
*L*3	6 m
*L*4	6 m
Gap mean height (*h*1)	0.5 m
Amplitude of the gap sinusoid (*h*2)	0.2 m
Beam cross-section (external diameter)	0.4 m
Beam cross-section (internal diameter)	0.3 m
Beam material elastic modulus	$10^{10}\;N/m^{2}$
Distributed load (*w*)	2500 N/m
Foundation parameter *k*0	$60,000\;N/m^{3}$
Foundation parameter *n*	2

**Table 6:** Beam and foundation parameters for Example 6.

Parameter	Value
*L*	6.096 m
Flexural rigidity (*EI*)	$8.18\times 10^{6}\;Nm^{2}\;\;$
*P*	133.4 kN
*w*	1.75 kN/m

## Data Availability

Some or all data, models, or code that support the findings of this study are available from the corresponding author upon reasonable request.
